# Ectopic Overexpression of an AUXIN/INDOLE-3-ACETIC ACID (*Aux/IAA*) Gene *OsIAA4* in Rice Induces Morphological Changes and Reduces Responsiveness to Auxin

**DOI:** 10.3390/ijms140713645

**Published:** 2013-06-28

**Authors:** Yaling Song, Zeng-Fu Xu

**Affiliations:** Key Laboratory of Tropical Plant Resource and Sustainable Use, Xishuangbanna Tropical Botanical Garden, Chinese Academy of Sciences, Mengla 666303, Yunnan, China

**Keywords:** Aux/IAA, 2,4-dichlorophenoxyacetic acid, gravity response, transgenic rice, tiller angle

## Abstract

Auxin has pleiotropic effects on plant growth and development. AUXIN/INDOLE-3-ACETIC ACID (Aux/IAA) proteins are short-lived transcriptional regulators that mediate auxin responses through interaction with an auxin receptor, the F-box protein transport inhibitor response 1 (TIR1). Most functions of Aux/IAA proteins have been identified in *Arabidopsis* by studying the gain-of-function mutants in domain II. In this study, we isolated and identified an Aux/IAA protein gene from rice, *OsIAA4*, whose protein contains a dominant mutation-type domain II. *OsIAA4* has very low expression in the entire life cycle of rice. *OsIAA4*-overexpressing rice plants show dwarfism, increased tiller angles, reduced gravity response, and are less sensitive to synthetic auxin 2,4-dichlorophenoxyacetic acid (2,4-D).

## 1. Introduction

It is known that auxin plays a very important role in a wide variety of plant developmental and physiological processes [1]. Several genes involved in the regulation of auxin dependent transcription have been identified, and of this Aux/IAA protein family members have been studied most thoroughly. Aux/IAAs are short-lived transcription factors comprising four highly conserved domains, known as domain I, II, III, and IV. Domain I contains conserved motif “LxLxL” and acts as a strong transcriptional repressor [2]. Domain II contains a core sequence “GWPPV” responsible for the rapid degradation of Aux/IAA proteins by interacting with a component of the ubiquitin-proteasome protein degradation pathway, which is stimulated by auxin [3,4]. Domains III and IV, shared by ARF proteins and Aux/IAA proteins, mediate homo- and hetero-dimerization among members of these two protein families [5,6].

Several Aux/IAA proteins with a mutation in domain II have been identified in *Arabidopsis*, including *IAA1/AXR5*, *IAA3/SHY2*, *IAA6/SHY1*, *IAA7/AXR2*, *IAA12/BDL*, *IAA14/SLR*, *IAA17/AXR3*, *IAA18*, *IAA19/MSG2*, and *IAA28* [7–17]. Several *Aux/IAA* genes were also identified in rice. *mOsIAA3*, a mutant with an amino acid changed in domain II, showed a gain-of-function phenotype like insensitive to auxin and gravitropic stimuli, exhibited short leaf blades, reduced crown root formation, and abnormal leaf formation [18]. *OsIAA13* is involved in lateral root initiation [19]. *OsIAA11* affected lateral root development [20]. *OsIAA23* is involved in postembryonic maintenance of quiescent center [21]. Our previous analyses of the expression profile of the *OsIAA* gene family in the developmental process and responses to phytohormones and stress treatments showed that most *OsIAAs* are involved in multiple hormone and abiotic stress responses [22]. *OsIAA1* is also involved in plant morphogenesis mediated by auxin and brassinosteroid (BR) hormone signaling pathway [23]. Unlike most *OsIAA* genes, which have high expression levels or organ/tissue expression patterns in the entire life cycle of rice, and are rapidly induced by auxin, the expression of *OsIAA4* (LOC_Os01g18360) is relatively stable and has a very low expression level in the whole life cycle of rice [22]. In this study, we further characterized *OsIAA4*, which has a partially conserved domain II, and contains an amino acid substitution that could cause a dominant mutation in *Aux/IAA* genes. Transgenic rice plants of ectopically overexpressing *OsIAA4* exhibited dwarfism, increased tiller angle, resistant to synthetic auxinic herbicide 2,4-D and decreased gravity response.

## 2. Results

### 2.1. Identification of *OsIAA4*

**Figure 1:**
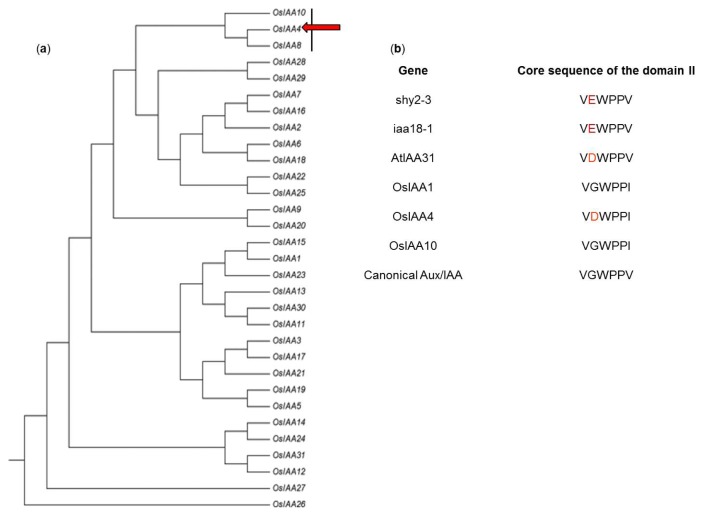
Identification of OsIAA4. (**a**) Phylogenetic tree of rice AUXIN/INDOLE-3- ACETIC ACID (Aux/IAA) proteins. Red arrow indicates position of OsIAA4 in the phylogenetic tree; (**b**) Domain II of different *Aux/IAA* proteins. Red letters indicate differences in domain II between dominant mutants (shy2-3 and iaa18-1) or non-canonical Aux/IAA (AtIAA31 and OsIAA4) and canonical Aux/IAA proteins.

The *OsIAA* gene family is involved in various developmental processes and responds to distinct phytohormones and abiotic stresses. Among them, *OsIAA4* and *OsIAA8* belong to one category with the expression level undetectable in almost all the organs/tissues [22]. Phylogenetic analysis of the *OsIAA* gene family shows *OsIAA4*, *OsIAA8*, and *OsIAA10* belong to one subclade, of which no further investigation into their functions have been reported (Figure 1a). Expression data from the CREB database (http://crep.ncpgr.cn/) [24] indicated *OsIAA4* has very low expression in the callus, stem, and endosperm, and no expression can be detected in any other organs/tissues. We were only able to obtain the full length cDNA of *OsIAA4* from the callus by RT-PCR, which confirmed the CREB data (data not shown). Sequencing results show *OsIAA4* encodes a 204-amino acid protein which contains four domains. In *Arabidopsis*, shy2-3 and iaa18-1 have a dominant mutation-type domain II that contains a G-to-E substitution; and AtIAA31 contains a G-to-D substitution in domain II that would cause a dominant mutation of *Aux/IAA* genes. In rice, OsIAA4 contains the same domain II as AtIAA31, whose core amino-acid motif is “DWPPV/I”, while the core motif of domain II of the canonical Aux/IAA protein is “GWPPV”, thus, OsIAA4 can be regarded as a dominant-mutation type protein (Figure 1b).

### 2.2. Morphological Changes of *OsIAA4*-Overexpressed Rice Plants

**Figure 2:**
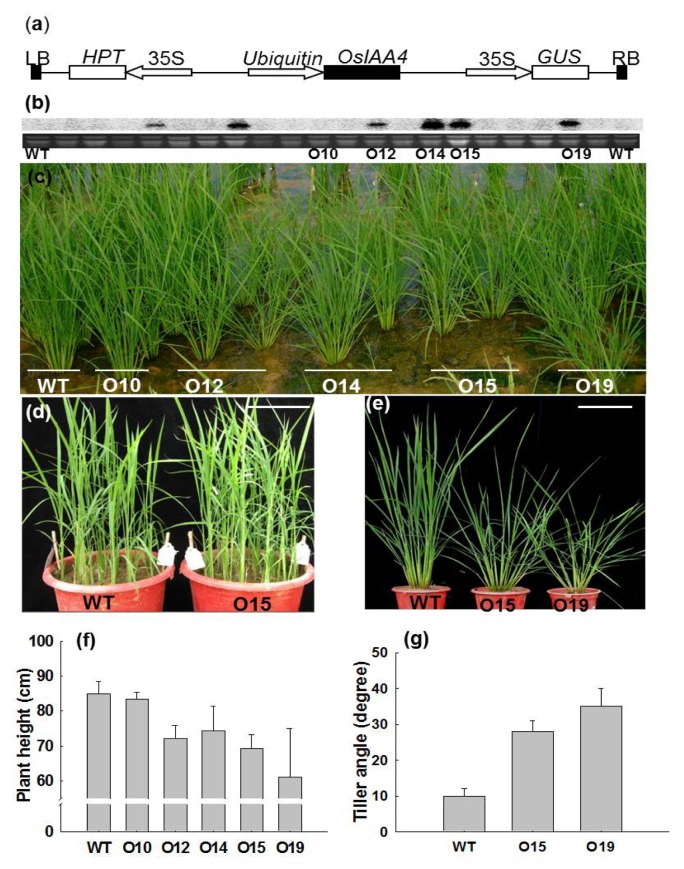
Morphological changes of *OsIAA4*-overexpressed plants under normal growth conditions. (**a**) Over-expression construct for rice transformation; (**b**) RNA gel-blot analysis of the transgenic and wild-type plants; (**c**) Vegetative growth of wild-type, non-overexpressing transgenic line (O10) and *OsIAA4*-overexpressed transgenic lines (O12, O14, O15 and O19) in field. *scale bar* in c = 9.5 cm; (**d**) WT and *OsIAA4*-overexpressed (O15) line at seedling stage. *scale bar* in d = 17 cm; (**e**) Increased tiller angle and dwarfism of over-expressed plants (O15 and O19) compared to the wild-type plants; (**f**) Quantification of plant height; (**g**) Quantification of tiller angle. Error bars indicate SD, *n =* 20.

To characterize the function of *OsIAA4* in rice, the full-length cDNA of *OsIAA4* was cloned into pCAMBIA1301U driven by a maize *Ubiquitin* promoter (Figure 2a) and transformed into Zhonghua 11 using the *Agrobacterium* method. In total, 20 independent *OsIAA4* transgenic plants were generated. Leaves of all the *OsIAA4* transgenic plants were collected for detecting the expression level of *OsIAA4*. RNA-blot analyses indicated that 30% of *OsIAA4* transgenic plants (6 lines) had higher expression levels compared to the WT (Figure 2b). To investigate the phenotypes of *OsIAA4*-overexpressing transgenic plants, four independent overexpression lines (O12, O14, O15, and O19) and one non-overexpressing line (O10) were grown in the field. At the seedling stage, no visible phenotype change was observed (Figure 2d). However, at the vegetative stage, the overexpression lines of *OsIAA4* showed significantly reduced plant height and increased tiller angle (Figure 2c,e). The height of *OsIAA4*-overexpressing plants is around 68 ± 5 cm while the control is around 85 cm (Figure 2f). The maximum tiller angle (angle between the main culm and its side tillers) in *OsIAA4*-overexpressing plants is more than 30 degrees but only 10 degrees in control plants (Figure 2g). The *GUS* gene-specific primers were used to check the positive transgenic plants and the PCR result indicated that the phenotypic change was co-segregated with *GUS* gene expression (data not shown). This result was obtained in T1 generation and similar result was repeated in T2 generation.

### 2.3. *OsIAA4*-Overexpressing Plants Show Less Sensitive to Auxin

**Figure 3:**
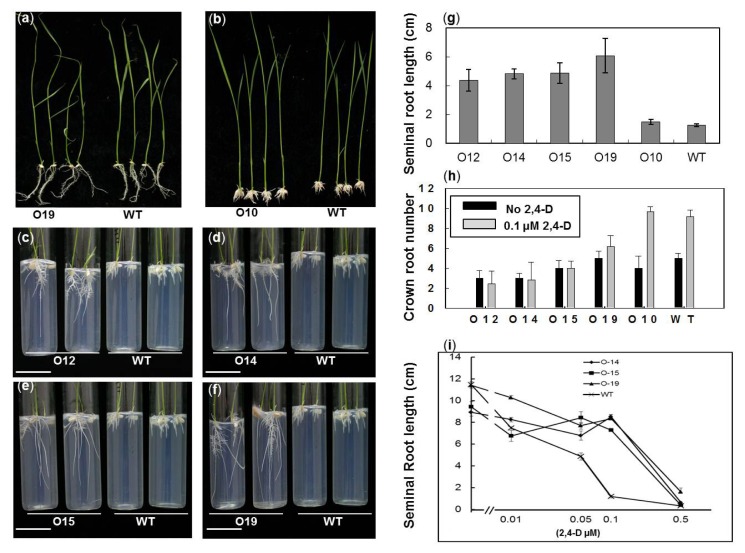
Root elongation assay of *OsIAA4*-overexpressing plants under 2,4-D treatment. (**a**) Transgenic plants and wild-type (WT) Zhonghua 11 grown in normal Murashige and Skoog (MS) medium; (**b**) non-overexpressing line (O10) and WT plants grown in MS medium with 0.1 μM 2,4-D; (**c**–**f**) Transgenic (O12, O14, O15, and O19), non-overexpressing transgenic (O10), and WT plants grown in MS medium with 0.1 μM 2,4-D; (**g**) Quantification of seminal root length in (c–f) (*t*-test, *p* < 0.01). *scale bars* in c–f = 29 mm; (**h**) Quantification of crown root number of transgenic (O12, O14, O15, and O19), non-overexpressing transgenic (O10), and WT plants grown in MS medium with or without 0.1 μM 2,4-D; (**i**) Dose responses of *OsIAA4*-overexpression (O14, O15, and O19) and WT plants to 2,4-D. Error bars indicate SE, *n =* 20.

To assess auxin responsiveness in *OsIAA4*-overexpressing plants, we performed auxin-responsive root elongation assays. Positive transgenic seeds were selected by germinating on hygromycin-containing Murashige and Skoog (MS) medium. Because of their faster growth rate, non-overexpressing transgenic and WT seeds were germinated on the MS medium one day later so that all germinated seeds were of a similar degree of vigor (with shoot length about 2 mm) for auxin treatment. Seedlings of similar height were transferred to MS medium containing 0.1 μM 2,4-D, and germinated seeds with the same vigor were transplanted onto the normal MS medium as a control. Two weeks after transplantation, there was no significant difference in plant morphology between the over-expressed plants and the control grown under normal conditions (Figure 3a) or the non-overexpressing transgenic line (O10) and WT growing in MS medium containing 0.1 μM 2,4-D (Figure 3b). However, the root length of the *OsIAA4* transgenic plant was significantly longer and the crown root number is significantly less than that of WT under 2,4-D treatment (Figure 3c–f). Auxin has inhibitory and promoting effects on root elongation and number of crown roots, respectively. These results show that *OsIAA4*-overexpressing plants were less sensitive to the inhibitory effect of auxin than the WT. Detailed data, including seminal root length were collected under 0.1 μM 2,4-D treatment (Figure 3g), and crown root numbers were collected under 0 μM or 0.1 μM 2,4-D treatment (Figure 3h). To further investigate the resistance to auxin response of transgenic plants, we examined the dose responses of *OsIAA4*-overexpressing plants to 2,4-D. The germinated seeds were transferred to MS medium containing 0, 0.01, 0.05, 0.1 and 0.5 μM 2,4-D and root lengths measured after two weeks of growth. The root length of transgenic lines (O14, O15, and O19) were significantly longer than the WT (Figure 3i), further confirming *OsIAA4*-overexpressing plants are less sensitive to the inhibitory effect of auxin than WT.

**Figure 4:**
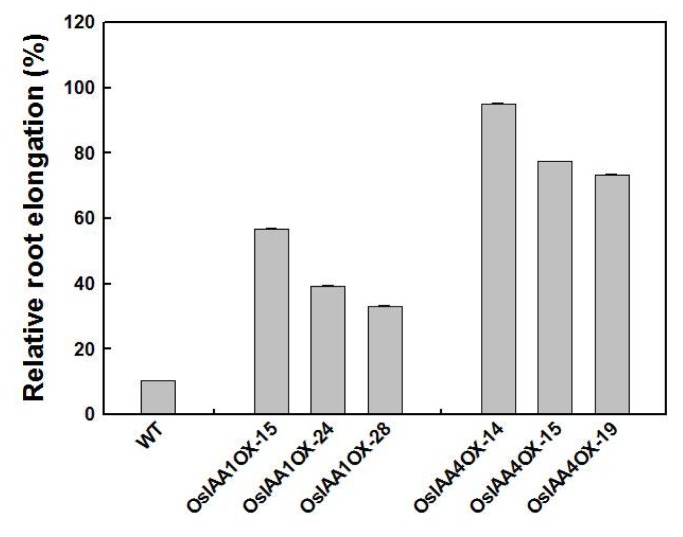
2,4-D effects on root elongation. Plants were grown in MS medium with or without 0.1 μM 2, 4-D. Root length was measured after 12 days. Relative root lengths are given as the percentage of root length of untreated plants grown in MS medium without 2,4-D. Error bars indicate SE, *n =* 20.

We also compared the auxin sensitivity between *OsIAA1*-overexperssing and *OsIAA4*-overexpressing plants by comparison of the seminal root length. Result shows *OsIAA4* transgenic plants displayed significantly stronger auxin insensitivity phenotype than *OsIAA1* transgenic plants (Figure 4).

**Figure 5:**
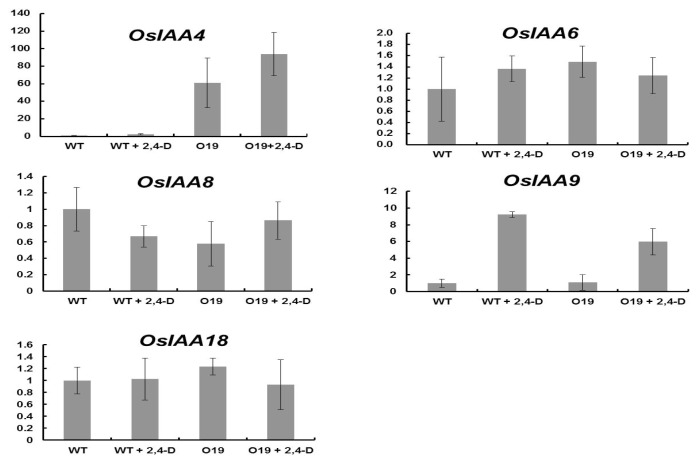
Real-time PCR analysis of transcript levels of auxin-responsive genes. The 8-day-old OsIAA4-overexpressing and WT plants were grown on normal MS medium or treated with auxin (20 μM 2,4-D, sampled at 3 h). The relative expression level of each gene was normalized to the expression level of the gene in the WT under normal conditions.

It is known that auxin signaling transduction has a negative feedback loop and Aux/IAA proteins function as a primary negative feedback regulator to auxin [25,26]. To test the role of non-canonical *OsIAA4* in this feedback pathway, the expression responses of five *OsIAA* genes, including *OsIAA8* (belonging to the same subclade as OsIAA4, which lacks domain II as do AtIAA20, 30, 32, 33, and AtIAA34), *OsIAA9* (showing a strong auxin up-regulation), and a pair of sister genes *OsIAA6* and *OsIAA18* which were significantly induced by drought [22] to auxin treatment were checked. We found that the expression responses of the *OsIAA* genes were not significantly changed in *OsIAA4*-overexpressing plants (Figure 5), suggesting *OsIAA4* is not the main auxin negative feedback regulator.

### 2.4. Overexpression of OsIAA4 Impaired Gravity Response in Shoot

**Figure 6:**
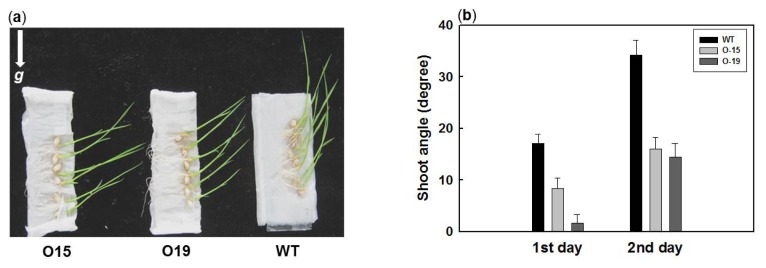
Gravitropism analysis of the *OsIAA4*-overexpressing and wild-type plants. (**a**) Three-day-old seedlings of *OsIAA4*-overexpressing (O15 and O19) and WT plants were grown vertically for one day and then horizontally grown for another two days. The arrow indicates the direction of gravity; (**b**) The shoot angle of WT and *OsIAA4*-overexpressed (O15 and O19) plants on the first day and second day. Values are means ± SE.

Gravitropic response is a typical auxin-related phenotype. Several Aux/IAA mutants display agravitropism in the hypocotyls and/or roots in *Arabidopsis*. In rice *lazy1* and *lpa1* mutant exhibits a tiller-spreading phenotype resulting from reduced shoot gravitropism [27–29]. Since *OsIAA4* transgenic plant shows typical auxin-related response and tiller-spreading phenotype, we assessed the gravitropic response of *OsIAA4*-overexpressing seedlings. We grew transgenic and wild-type seedlings vertically for one day, and then reoriented them by 90 degrees (horizontally) for an additional two days and measured the angle of shoots. We found that *OsIAA4* transgenic plants displayed impaired gravitropic responses compared to the WT (Figure 6a). The shoot angles of transgenic plants (25 degrees) were significantly smaller than those of the WT plants (35 degrees) on the second day of reorienting (Figure 6b).

## 3. Discussion

It is well known that rapid turnover of Aux/IAA protein is required for normal auxin response and domain II contains a “qvVGWPPvrsyRkN” conserved motif; in particular, the conserved “GWPP” has been linked to rapid degradation in canonical Aux/IAA family members. Mutation in domain II results in relative stabilization of the mutated proteins. Several gain-of-function mutants with altered auxin response or morphology have been identified in *Aux/IAA* genes in *Arabidopsis*. These auxin responses and morphologies include phototropism/gravitropism, root formation, apical dominance, stem/hypocotyl elongation, and leaf expansion. In an earlier *OsIAA* family analysis, *OsIAA4* was found to belong to one small subgroup that has very low expression levels in the whole life cycle of rice [22]. Phylogenetic tree analysis indicated OsIAA4, OsIAA8, and OsIAA10 form one small subclade. Predicted protein sequence showed that the conserved G in domain II is replaced to D in OsIAA4. Ectopic overexpression of *OsIAA4* shows dwarfism, increased tiller angle, insensitivity to the inhibitory effect of auxin on root elongation, and impaired gravity response in shoots. IAA31 has the same domain II as OsIAA4 which cause a dominant mutation of *Aux/IAA* gene in *Arabidopsis*. Overexpression of IAA31 in *Arabidopsis* by using cauliflower mosaic virus 35S promoter exhibited auxin-related aberrant phenotypes including smaller plant size, longer lateral stems than primary stems, and defects in gravitropic response in hypocotyls [30]. All these results suggested that ectopic overexpression of dominant mutation-type Aux/IAA proteins cause auxin-insensitive related phenotypes.

Auxin plays a crucial role in root development including regulation of the main root growth and the number of lateral, adventitious roots in *Arabidopsis* [8,12,13,17]. Auxin-insensitive phenotypes such as dwarfism, decreased gravitropic response, and suppression of root growth were observed by studying mutants in *Arabidopsis*. These abnormal phenotypes were also found in *OsIAA1*-overexpressing and *mOsIAA3* plants [18,23]. In *OsIAA4*-overexpressing plants the growth of the seminal root was insensitive to 2,4-D and the number of crown root was decreased under 2,4-D treatment, suggesting auxin contributes to the root development in rice similar to its effect in *Arabidopsis*.

*Aux/IAA* is a large gene family with more than 30 members in rice. In earlier work we identified several T-DNA insertion mutants of *OsIAAs*, and no obvious changes in morphology or hormone responses were observed, implying functional redundancy [22]. The protein sequence of OsIAA4 shows it has a dominant mutation-type domain II, which contains “DWPPI” instead of “GWPPV/I”. As no T-DNA insertion mutant of *OsIAA4* was identified, we made *OsIAA4-*RNAi transgenic plants, but found no visible phenotypic change (data not shown). Over-expression of *OsIAA4* shows a similar auxin response to over-expression of *OsIAA1*. Both *OsIAA4* and *OsIAA1* exhibited decreased auxin response, but the *OsIAA4* transgenic plants displayed a stronger auxin insensitivity phenotype, possibly associated with the longer half-life of OsIAA4; OsIAA1 contains intact domain II while OsIAA4 contains a dominant mutation-type domain II. Instead of an increased lamina joint angle, as in the *OsIAA1* transgenic plant, *OsIAA4* exhibits increased tiller angle, suggesting each *OsIAA* may have a specific function in its developmental process. These results together suggest *OsIAA1* and *OsIAA4* are functionally redundant and specific.

Rice is one of the most important food crops and is a model monocot. Rice yield is mainly modulated by its architecture [31,32], which is defined by tiller number and angle, internodes elongation, panicle morphology and leaf angle [33,34]. Tiller angle is defined as the angle between the main culm and its side tillers, and is critical for dense planting because a wide tiller angle will increase leaf shade and decrease photosynthesis efficiency, whereas a narrow tiller angle is more efficient in plant architecture [35]. Leaf angle is the inclination between the leaf blade and vertical culm which is mainly controlled by the leaf lamina joint [36]. Over-expression of *OsIAA1* changed the leaf angle and over-expression of *OsIAA4* changed the tiller angle, which may be useful for molecular breeders to achieve ideal plant architecture to improve grain yield. Rice plants develop tillers from the axillary buds. The roles of auxin in the development of axillary buds in *Arabidopsis* imply that auxin and its signaling may be the key regulator in the development of tillers. Transgenic plants with a reduction of *OsPIN1* gene expression display an increase in tiller number as well as tiller angle [37]. It is known that auxin transport is mainly controlled by auxin influx (AUX1) and the PIN-FORMED (PIN) efflux carriers and the MULTIDRUG RESISTANCE/P-GLYCOPROTEIN (PGP) class of ATP-binding cassette auxin transporters. *Aux/IAAs* are early auxin responsive genes. It is not surprising that *OsIAA4*-overexpressing plants have a similar phenotype to *OsPIN1* down-regulated plants. Although two different types of auxin related genes were found to be involved in rice tiller angle (*OsPIN1* and *OsIAA4*), the underlying molecular mechanism of how these two types of genes control rice tiller development is still unknown. Further work should focus on deciphering the precise molecular mechanism on how *OsPIN1* down-regulated plants show similar phenotypes to *OsIAA4* overexpression plants.

## 4. Materials and Methods

### 4.1. Construct and Rice Transformation

The full-length cDNA of *OsIAA4* was amplified from Minghui 63 (*Oryza sativa* L. ssp. *indica*) callus using primer: 5′-ATAGGTACCTATGGAGGAGTGCAAGGG-3′ and 5′-TATGGATCCTTCTG CTATACGGTAGGTAG-3′. Sequence was obtained by RT-PCR and confirmed by sequencing, and digested by *Kpn*I and *Bam*HI and then inserted into pCAMBIA1301U under the control of a maize ubiquitin promoter, and transferred into Zhonghua 11, japonica rice that can be easily transformed by *Agrobacterium*-mediated transformation method.

### 4.2. Northern Blot and Real-Time PCR

Total RNA was isolated from T0 transgenic plants with Trizol reagent (Invitrogen, Carlsbad, CA, USA) according to the manufacturer’s instructions. Total RNA was separated on a 1.2% agarose gel containing 2% formaldehyde and then transferred onto a nylon membrane by capillary method. RNA gel blots were hybridized with ^32^P-dCTP-labeled gene-specific probes over-night using Perfect-HYB Plus buffer (Sigma, St. Louis, MO, USA) at 65 °C. Blots were washed three times (twice with 2× SSC/0.1% SDS for 10 min and once with 0.5× SSC/0.1% SDS for 5 min) at 65 °C, and then subjected to radiophotography.

For real-time PCR analysis, first-strand cDNAs were synthesized from DNaseI-treated total RNA using Superscript II reverse transcriptase (Invitrogen) according to the manufacturer’s instructions. Real-time PCR was performed in an optical 96-well plate with an ABI PRISM 7500 real-time PCR system (Applied Biosystems, Foster City, CA, USA). Each reaction contained 12.5 μL of SYBR Green Master Mix reagent (Applied Biosystems), 4.0 μL of cDNA samples, and 200 nM gene-specific primers in a final volume of 25 μL. The thermal cycle used was as follows: 95 °C for 3 min; 45 cycles at 95 °C for 30 s, 60 °C for 30 s, and 72 °C for 1 min. The *Osactin* gene was amplified as an internal control to quantify the relative amounts of cDNA. The primers used for real-time PCR are listed in Table 1.

### 4.3. Plant Growth and Treatments

Transgenic seeds were germinated on a Murashige and Skoog (MS) medium with 50 mg/L hygromycin and wild-type (WT) seeds were germinated on a MS medium (usually one day later) without hygromycin at 26 °C. Plantlets with same vigor (shoot length 2–3 mm) were transferred onto the MS medium containing different concentrations of 2,4-D and transferred to a growth chamber at 26 °C with a 14-h-light/10-h-dark cycle. Plants were measured for root length and crown root numbers were counted 12 days after transplanting. For the gravitropic response experiment: transgenic and wild-type seeds were germinated on a MS medium, three-day-old plants were grown vertically for one day and reoriented by 90° and grown for another two days. The shoot angle was measured. To check the expression levels of auxin responsive genes in the *OsIAA4* overexpression plants, 8-day-old transgenic and wild type (WT) seedlings were treated with 20 μM 2,4-D and sampled at 3 h.

## 5. Conclusions

In conclusion, our results indicate *OsIAA4* is a low expression gene, encodes an Aux/IAA protein containing a dominant mutation-type domain II, and *OsIAA4* overexpression lines have typical auxin related characterization. Overexpression of *OsIAA4* resulted in dwarfism, increased tiller angle and decreased gravity response.

## Figures and Tables

**Table 1:** Primers used for real-time PCR.

Gene	Forward primer	Reverse primer
*OsIAA4*	GCTCTTGCTGGATGGGTATGA	AGGTGATGGGCGTCTTGAAC
*OsIAA6*	GGCTATCGTCAGCTGTCAAACA	GCAATTTGCGCATTAGTTTGG
*OsIAA8*	CCGCTAGACGGCTACAAAGG	GGTGATGGATGCTCTGAACATG
*OsIAA9*	CGAGAAGAAAATGGCCAATGA	ATCCCCATCACCATCCTCGTA
*OsIAA18*	AAGAATGTGGGAAGGAGCTAACG	ATGGTGGTGAGGGACAGCAT
*Osactin*	TGGCATCTCTCAGCACATTCC	TGCACAATGGATGGGTCAGA
